# Institutional dynamics and learning networks

**DOI:** 10.1371/journal.pone.0267688

**Published:** 2022-05-16

**Authors:** Philip Poon, Jessica C. Flack, David C. Krakauer

**Affiliations:** Santa Fe Institute, Santa Fe, NM, United States of America; Indiana University, UNITED STATES

## Abstract

Institutions have been described as ‘the humanly devised constraints that structure political, economic, and social interactions.’ This broad definition of institutions spans social norms, laws, companies, and even scientific theories. We describe a non-equilibrium, multi-scale learning framework supporting institutional quasi-stationarity, periodicity, and switching. Individuals collectively construct ledgers constituting institutions. Agents read only a part of the ledger–positive and negative opinions of an institution—its “public position” whose value biases one agent’s preferences over those of rivals. These positions encode collective perception and action relating to laws, the power of parties in political office, and advocacy for scientific theories. We consider a diversity of complex temporal phenomena in the history of social and research culture (e.g. scientific revolutions) and provide a new explanation for ubiquitous cultural resistance to change and novelty–a systemic endowment effect through hysteresis.

## Introduction

Two notable characteristics of cultural life are stasis and transformation. Braudel described the heterogeneous movements of history as ‘what moves rapidly, what moves slowly, and what appears not to move at all’ [1]. Over the course of years, some patterns of belief, scientific theories, fashions, firms, and political views are fickle whereas others appear frozen. In due course all institutions are subject to change, often precipitous. What causes institutional change and what might account for variation in the rate of change? Can we answer the question behind the celebrated quote inspired by Lenin, ‘There are decades where nothing happens; and there are weeks where decades happen’?

The idea of the “institution”, defined very generally as the humanly devised constraints that structure political, economic, and social interactions [2], includes a range of diverse phenomena [3] from social norms and laws to firms, political doctrines, and scientific theories. This space can be organized into institutions supported by codified laws and policies including firms, markets, and rule-based abstractions such as scientific theories, fashions, political beliefs, and social norms encoded largely through collective perception [4–7]. These degrees of institutional structure are related over time as collective perceptions and beliefs become codified. In this paper, we develop a novel, multi-scale modeling framework to study how such collectively encoded institutions change.

When examining empirical examples of institutional change three types of *surface* or visible change stand out. In the first category are forms of institutional change that appear to exhibit long periods of stasis, even active resistance to change, and thereafter experience a rapid switch of belief. In the field of astronomy the geometric theories of Ptolemy—bolstered by the ideology and power of the church—prevailed over all regularities of planetary motion for over one and a half millennia. Evidence complicating the Ptolemaic model was not sufficient to lead to its abandonment until the contributions of Brahe, Galileo and Kepler, supported by a variety of collective institutions to include the Royal Court of Denmark [8], the Medici family [9], and the Academy of the Lynx [10], lead to its overthrow. The theory of spontaneous generation was an accepted mechanism accounting for microbial life for over two millennia, maintained in part by the authority of Aristotle and his adherents–even in the face of the ingenious counter evidence from Francesco Redi. The idea was eventually accepted as overturned but it took falsification by Pasteur with support from the French Academy of Sciences. Within a few years of this support the majority or biologists rejected the idea [11]. Similarly, the theory of plate tectonics, first proposed in 1915 was ignored by many and contested until the mid 1960s. By 1970, within less than a decade, it was the dominant theory accounting for the structure and distribution of the continents [12, 13].

By contrast, other collectively encoded institutions exhibit persistent volatility [14]. Examples from this second category of surface change include the hemline [15] for fashion, political preferences and changing support for U.S. political parties [14], as well attitudes toward the filibuster [16], which has been tracked since Gallup’s first poll on the topic in 1937. Support for the death penalty has a similar character and switched very briefly to a “majority against” position in the 1960’s only to return to a “majority for” position shortly afterwards [17]. Thereby showing a tipping-point characteristic.

Finally surface change can also be gradual, such that it is hard to say when–or sometimes even if–a change in an institution has occurred. An example of an institution that has shown gradual surface change is the political doctrine of liberalism [18]. Liberalism has morphed from its introduction in the nineteenth century as a somewhat elitist notion of government by “the best” towards a laissez-faire, free market conception of rights, towards most recently an idea closer to social democracy. Throughout its evolution it has retained some notion of individual duties or rights at its core.

These descriptive characterizations based on rates of surface change often fail to capture change progressing in underlying, largely invisible, dynamics. A recent study of support for gay marriage in the United States found many individuals had reversed their position on gay marriage from against to for but were not expressing their attitudes because they perceived the majority to be against it. Support for gay marriage had in fact *accumulated* but remained latent because of a signaling problem that was ameliorated by top down support—the passing of legislation in support of gay marriage [19]. This demonstrates how the surface rate of change–which reflects expressed perceptions only–can obscure underlying dynamics. In order to understand the causes of change, mechanism must be considered. In particular, how individuals *read* and *influence* the *collective view* [20].

Kuhn’s The Structure of Scientific Revolutions is perhaps the best known framework for explaining change that extends beyond individual discoveries to collectives [21]. Evidence is accumulated within ‘paradigms’ that modulate the impact of individual inventions. In periods of ‘normal science’ a given paradigm dominates the interpretation of all results—corresponding to stasis in belief. ‘Paradigm shifts’ occur once sufficient evidence has accumulated supporting an alternative model. However, the volatility and gradual change examples suggest other types of surface change than Kuhn stressed, and we want a modeling framework that not only can accommodate these other forms, but also distinguishes between surface change and underlying dynamics.

We operationalize the current socioeconomic definition of institutions–’recognized patterns of behavior that define, govern, and constrain action’–to encompass the idea of scientific ‘paradigms’, in terms of two institutional properties: (1) visible “public position” scores derived from (2) ledgers that encode a full history of collective opinions. The ‘institution as ledger’ framework integrates both positive and negative votes into the single, summed, public position. The ledger and public position concepts allows us to assign quantitative popularity scores to what is otherwise a qualitative idea. In this paper we model the coarse-grained history of support and opposition through their influence on visible public positions.

Using dynamical systems we analyze the timescale of positional change influencing agent behavior. These are timescale upon which the collective view biases institutional changes. These changes can be relatively fast or slow, but with the possible exception of very small systems or those in which individuals are highly correlated, they will by construction have a slower rate of change than the individual opinions from which institutions emerge. The public positions are modified according to specific rule systems (i.e. learning rules that modify an agent’s ability to contribute position entries), and they feedback to influence future patterns of agent-interaction (by being ‘read’ by agents) [20].

Treating collective encoding as a public position in a ledger allows us to study how agents and institutions interact. The primary explanation for directed and cyclical change in human institutions is that agents are able to learn adaptively and modify their strategies over time to achieve short and long-term goals. This learning perspective pervades modern game theory [22], institutional economics [23], education and research [24], protest movements [25], modern social movements actuated by social media [26]. That being said, models and theory have tended toward equilibrium frameworks where strategy sets are fixed, or, when learning is considered, models dominated by interactions at the agent level. By contrast we are considering learning processes that span both agent interactions and agent-institution interactions. Agent-institution learning interactions, which entail agents perceiving and reading institutional positional values as well as constructing or ‘writing’ to them them, have been considered in the empirical and experimental literature(*e.g.* [20]) but rarely directly in strategic dynamics. In the empirical literature issues relating to trends, cyclicality, switching, and the appearance of stasis (quasi-stationarity) have been discussed in a variety of contexts from habit-formation [27], norm-enforcement [28], and the opposition to new scientific models and theories [11, 13].

In order to capture empirical patterns of individual and institutional change we consider a dynamical system organized into a bipartite hierarchical network–with competing agents at one level learning to construct, or write to, institutions which act as ledgers whose public expressions are public positions that can be accessed or ‘read’ by populations of agents. These positions when read provide agents with differential competitive advantages by biasing their preferences and generating a herding effect at the population level. A key feature of learning networks is that they evolve at multiple time scales: a slow time scale for position values and a faster time scale for agent preference and, in doing so, serve as a stable, collective property of the environment that agents can “read” in order to estimate the aggregate opinions of other individuals. This approach is related to niche construction theory [29, 30] and effective downward causation arising through collective coarse-graining [20]. Through a non-equilibrium, slow manifold theory, we analyze the origins of both quasi-stationary (the appearance of stasis), one-time switching (a single flip in the dominance/abundance of agents), and periodically evolving organizations (ongoing cycling in the abundance of agents). Elimination of one or more assumptions of the model (e.g. zero learning or constant entries for the position values) recovers simpler game theoretic approaches to institutions where payoffs are constant, interactions are zero sum, and fixed point Nash equilibria can be reached.

## Materials and methods

### A Framework for learning institutions

**Fig 1:**
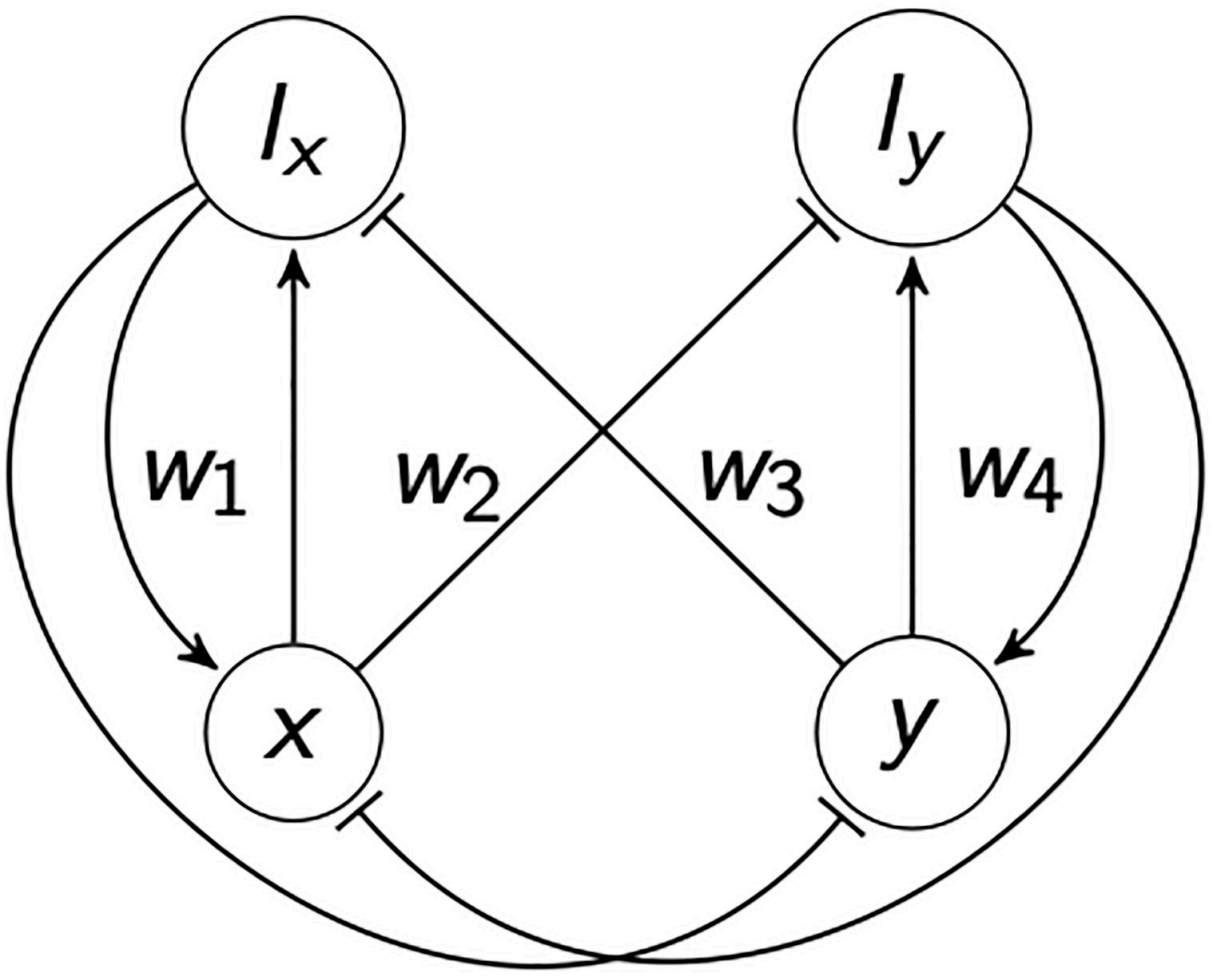
The feed-forward and feed-back structure of populations of agents (*x*, *y*) competing through learned (*w*) support for two institutions that act as ledgers summing inputs into position values (*I*_*x*_, *I*_*y*_) that quantify power or popularity. These values are therefore the sum of a history of popularity and provide feedback by being ‘read’ by populations of agents. Individual agents change in abundance quickly, whereas institutions—as captured in these position values—change slowly through slow learning processes. Standard arrows on edges encode excitation and perpendicular bars on edges encode inhibition. Learning rules operate on the values of *w* modifying the amplitude of excitation and inhibition.

The basic framework is set out in Fig 1. Here we justify the elements: interactions, dynamics, and learning rules, that will be put into a formal dynamical system in subsequent sections. We consider ideas or norms as transmissible and able to spread through a population by means of persuasion, coercion, and imitation. The spread of ideas will be captured through a model of contagion [31] in which two populations of agents *x* and *y* compete directly. We add novel terms to this population dynamic in order to allow for institutions that encode the sum of inputs like ledgers in summary public-position values *I*_*x*_ and *I*_*y*_ that bias the outcome of agent interactions. We assume that these positions encode real-valued sums of agent preferences generated through weights, or voting factors *w*. These weights map agent numbers into institutional popularity/social power—the real values encoding agent contributions to population dynamics through a “position value”. The weights are learned through a family of learning rules –see below– (and S7 File for the mono-polar case) that capture the success of agents at supporting or inhibiting institutional growth. As we shall show, ongoing learning tends to eliminates fixed points and leads to a ‘slow manifold’. This manifold describes variables that change slowly in time and that come to dominate the dynamics of the system.

To best illustrate how switching between agent populations might arise we consider the case where one agent or belief is locally more persuasive than another—that is where there are asymmetries in spreading. For example, populations of smokers *y* might convince populations of non-smokers *x* to take up smoking. And this behavior is then perpetuated through habit and addiction. The behavior of this basic model without institutions is analyzed in ‘S1 File’. As per intuition highly persuasive agents will dominate a population all else being equal. For example, smokers recruiting non-smokers by direct contact. But of course the spread of smoking is hugely amplified through institutions such as the advertising industry. Institutions s encode influence in public position values *I*_*y*_. And these institutional terms increase the persuasive power of *y* when encountering *x*. However non-smokers are not powerless. They can support institutional regulation and counter-campaigns that make their own public position values *I*_*x*_, such as publicly accessible laws and facts of health education. These will in turn limit the adoption of smoking and promote quitting. The ethical implications of shaping institutions of this form are obviously of considerable social significance.

This framework extends to the US two-party system. Like-minded citizens organize politically according to their beliefs. Highly ideological agents support their own institutions or parties by attending party conventions recording their support in numbers at rallies and aggressively countering the influence of their rivals through protesting. Political parties grow with the number of their supporters and simultaneously strive to limit numbers in opposition (without assuming that this interaction is zero sum in the payoff). Under this scenario one might expect a single, highly motivated party to dominate in the same way that smoking might be expected to take over non-smokers. The key to party political change lies in the way affiliated agents learn to support and oppose institutions through time. And the same bicameral logic can be extended to proponents of a new theory better able to account for observation (interpreted as the locally transmissible variable *y*) that is not able to fix or dominate belief as a result of institutional conflicts of interest (a single Institution model is explored in S7 File).

### Learning rules

A key to understanding any social change is how agents learn, both individually and collectively, and thereby contribute to strengthening allied institutions and diminishing rival ones. A variety of learning theories have been proposed to account for resistance to change at the individual level, to include extensions of behavioral momentum theory which posit an interplay between a behavioral mass (weight of learned experience) and the strength of reinforcement schedules [32]. Extending these insights to collectives is in its early days but can profitably be related to social cognition [33] and social contagion [34]. There have been a number of empirical case studies drawing on this learning literature exploring conditions favoring stasis versus change. These include learning of social norms by children observing norm-enforcement by parents [28], the role of signaling in communicating perception of a social rule in chimpanzees [7], the acquisition of political identities based on imitating trusted models [35], the role of institutions (exercising third party punishment) in promoting pro-sociality [36], and the value of advertising campaigns in promoting the learning of health-related behaviors (e.g. the cessation of smoking [27]). In all of these cases mechanisms of associative or aversive learning combined with a degree of behavioral momentum are assumed to underlay observed patterns of behavior.

**Fig 2:**
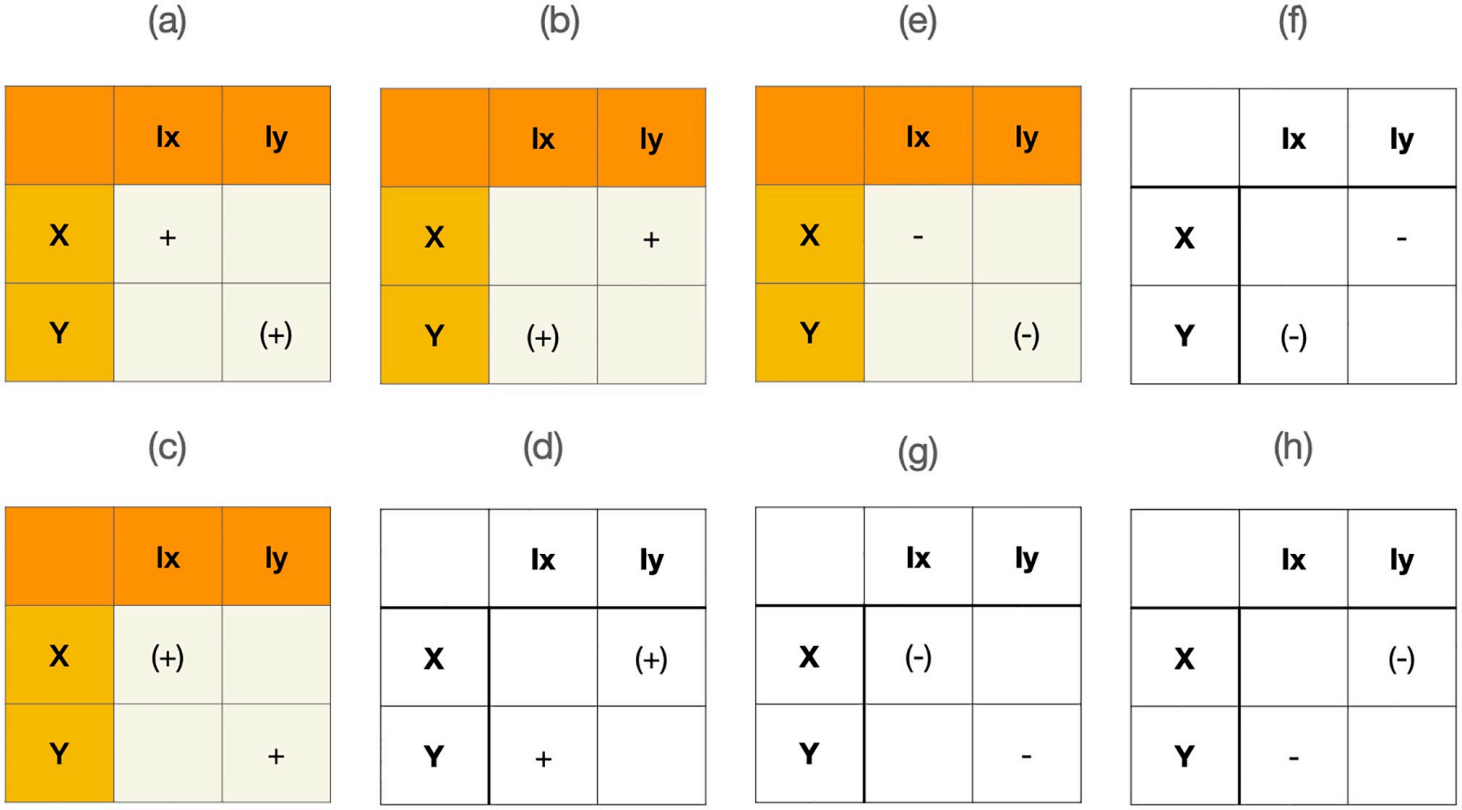
Classification of reinforcement learning rules. The sign of each the entries denotes positive or negative reinforcement acting on weights between a population of agents (*x*, *y*) and institutional position values (*I*_*x*_, *I*_*y*_). Consider (a) as an example: this rule describes positive reinforcement on weights mediating interactions between *x* and *I*_*x*_ or *y* and *I*_*y*_. Whereas rule (e) is the dual of rule (a) and only involves negative reinforcement–increases in the abundance of agents or position values leads to a reduction in their connecting weights. Generally naked signs +/- indicate the polarity of change to *w*_1_ (the sign to *w*_2_ is the negative of this sign) and parenthetical signs (+/-) are the polarity of the change to *w*_4_ (the sign to *w*_3_ is the negative of this sign). We explore the four rules shaded in orange which we call (a)—the Hebbian Rule; (b)—the Competitive Rule; (c)—the Compensation Rule; (e)—the Least Effort Rule. Rules (a,b,c,e) are all generated through successive 90° rotations starting from the identity matrix (a) and rules (d,f,g,h) through successive 90° rotations from (e).

We consider reinforcement learning and momentum at a collective level through four learning regimes that span a space of plausible institutional learning rules (S6 File). A Hebbian-like rule, a Least Effort rule, a Compensation rule, and a Competitive rule. See Fig 2). Values used in these plots can be found in S2 File. These four rules live in a reduced space of binary interactions. One can conceive of an expanded space of triplets or quadruplets where agents modify their response to the entire network representing global information. In this paper we consider local information defined by exclusively pairwise learning sets. Crucially, as we show, the choice of learning rule determines the ensuing institutional dynamics. This suggests that rule choice could act as a mechanism for emergently engineering institutional change.

The Hebbian rule is a mutually reinforcing rule generating positive returns whereby agents (*x*, *y*) interact directly with supporting institutions through position values (*I*_*x*_, *I*_*y*_) to improve their ability to support themselves. This rule provides positive feedback to each strategy in proportion to their abundance. Unlike canonical neural, Hebbian mechanisms, we do not need to assume strict temporal contiguity as reinforcement takes place across the whole population over time. Hebbian-like rules are those dominated by interactions between agents and their own favored institutions, as is often observed in the championing of athletes by agencies [37]. The Least Effort rule captures negative returns or learning through effort minimization. It is the negative of the Hebbian Rule. The more abundant a class of agents and the higher their position values, the less effort is invested in learning how to construct supporting institutions. The Compensation rule is responsive only to the success of rival agents and institutions. It follows the logic of an arms race whereby learning takes place only when rivals and their institutions are abundant. The Competitive rule is a revolutionary rule as agents learn to construct in proportion to their own abundance and the abundance of a rival institution. An empirical example of rules dominated by consideration of rivals is observed in highly imitative markets with extensive lobbying such as mobile phone technologies [38].

We also consider various combinations of these rules. Combining the Hebbian rule and the Compensation rule describes positive reinforcement that manages finite resources of time and energy. For example, in the domain of marketing [39], the Coca Cola company tends to follow a Hebbian rule (advertise in proportion to its own market dominance): ‘Three Million a Day’ (1913) ‘Coke Is It!’ (1982), whereas the Pepsi company favors a compensation rule (advertise in response to the strategy of its competitor Coca Cola): ‘Twice As Much For A Nickel Too’ (1939) and ‘Taste the difference (1985). These difference highlight Pepsi’s well known ‘niche strategy’ characterized by its dependence and response to the market leader (Coca Cola) [40]. The dynamical behavior of each combination of rules is enumerated in S5 File.

### Dynamics of complex time

Building on the fundamental idea of a multiplicity of functional time scales defining complex adaptive systems we model institutional change by embedding agents and institutions in a learning network with multiple time scales. This non-equilibrium, multiple timescale approach allows us to represent both agent behavior which tends to be fast captured as basic transmission process and modeled as an infectious dynamic in which a populations of agents *y* seek to influence a population of agents *x*, as well as the larger and slower institutional position values that they collectively construct. Since the institutions record the inputs of many agents they naturally encode a history of preferences.

$$\begin{array}{c} \frac{dx}{dt}\;=r-s\left(I_{y}+q\right)xy-\lambda_{x}x+cI_{x}y\;; \end{array}$$ (1)

$$\begin{array}{c} \frac{dy}{dt}\;=s\left(I_{y}+q\right)xy-\lambda_{y}y-cI_{x}y\;. \end{array}$$ (2)

Table 1 provides the definitions of the variables and parameters of the model. The effective persuasion of *y* on *x*, and the effective conversion of *x* and *y* are given by *s*(*I*_*y*_ + *q*), (λ_*x*_*x* − *cI*_*x*_*y*), and (λ_*y*_*y* + *cI*_*x*_*y*). The influence of the two institutions through their position values *I*_*x*_ and *I*_*y*_ is felt in both the multiplicative contact term featuring *xy* and in the growth term in *x* and decay term in *y*.

**Table 1:** Table of variables and parameters employed in institution construction model.

Variables	Definition
*x*, *y*	Populations of competing agents
*I*_*y*_	Value of public-position for Institutions constructed by *y* to support *y* and suppress *x*
*I*_*x*_	Value of public-position for Institutions constructed by *x* to support *x* and to suppress *y*
$\vec{w},\vec{\gamma }$	Learned weights associated with *x* or *y* in the construction of *I*_*x*_ or *I*_*y*_
Parameters	Definition
*r*	Migration rate parameter of *x*
λ_*x*,*y*_	Death rate parameter of *x* or *y*
*s*	Contagion/competitive impact rate parameter of *y* on *x*
*q*	Baseline impact rate parameter of *y* on *x*
*Q*	The quantitative impact of agents on their institutions
*c*	Scaling factor of *I*_*x*_ to the death rate parameter
*k*	Positive feedback rate parameter of $\vec{w}$ or $\vec{\gamma }$
*p*	Death rate parameter of $\vec{w}$ or $\vec{\gamma }$

The dynamics of *I*_*y*_ and *I*_*x*_ are described via the following fast construction rules that define a simple feed-forward neural network with weight parameters *w* quantifying the ability of each strategy to construct its supporting institution:

$$\begin{array}{c} I_{x}=\phi (w_{1}x-w_{3}y)\;; \end{array}$$ (3)

$$\begin{array}{c} I_{y}=\phi (w_{4}y-w_{2}x)\;. \end{array}$$ (4)

Where $\phi \left(z\right)=\frac{Q\text{exp}z}{\text{exp}z+Q}$. This exponential function is the familiar activation or squashing function of neural networks which constraints the values of *I*_*x*_ and *I*_*y*_ to lie in the range [0,Q]. This places a ceiling on the popularity or power of any institution. The exponential form is not critical for the dynamics only that *φ*(*z*) be a continuous function in the interval [0,Q]. The dynamics of these weights are governed by a class of slow learning rules that obey the following functional dependencies:

$$\begin{array}{c} \frac{dw_{i}}{dt}\;=kf_{i}\left(I_{x},I_{y},x,y\right)-pw_{i}, \end{array}$$ (5)

These rules, through a suitable choice of *f*_*i*_ (and the variable *g*_*i*_ in the three dimensional system to include the slow manifold in sections below), allow both *x* and *y* to build up *I*_*x*_ and *I*_*y*_ in order to compete amongst each other more effectively. For completeness we also consider the case where we allow for an explicit dynamics governing *I*_*x*_ and *I*_*y*_. In this way rather than completely separating the time scales of construction and learning we allow them to overlap. Under reasonable assumptions this modification has little effect on the qualitative results (see S3 File) and only adds a constant of proportionality to the analysis.

To expedite analysis assume symmetry of λ_*x*_ = λ_*y*_ = λ, and $\frac{r}{\lambda }=\hat{r}$ and thus $x\left(t\right)+y\left(t\right)=\hat{r}-O\left(\text{exp}\left(-\lambda t\right)\right)$. In other words, we bound the sum of *x* and *y* to $\hat{r}$. We note that for any adaptive weights *w*_*i*_ that have the same nonlinear term in *g*_*i*_, *w*_*i*_ − *w*_*j*_ = (*w*_*i*_(0) − *w*_*j*_(0))exp(−*τ*), *w*_*i*_ ‘and *w*_*j*_ collapse onto the same dynamical variable in exponential time in the slow time scale. Here *τ* is a new effective slow time scale (see derivation in S6 File). These results lead to a reduction in dimension and complexity of the model. We can then classify the models in terms of a number of effective dynamical variables and coupling among slow variables.

### Learning rules for adaptive weights

The key innovation in these models is the integration of learning rules into contagion dynamics. The dynamics of institutional learning, as illustrated in Fig 2 are captured by the dynamics of the weights $\vec{w}$ described by differential Eq (5) and the dynamics of construction by *f*_*i*_ (or *g*_*i*_). Institutions introduce two extra time scales into the model. Beyond the time scale of interacting agents there is the time required to support or construct an institution and the time required to learn how to effectively construct it. The learning time scale is the crucial one (see S4 File for further remarks on multiple timescales). These learning rule provide a classification of agent-institution relationships. Each of the learning rules can be classified according to the patterns of pair-wise interaction determining the form of *f*_*i*_ as enumerated in the following Table 2.

**Table 2:** The pairwise, multiplicative terms corresponding to each of the four learning rules.

	Hebbian	Least Effort	Compensation	Competition
*f*_1_	*I*_*x*_ *x*	*xy*	*I*_*y*_ *y*	*I*_*y*_ *x*
*f*_2_	*I*_*y*_ *x*	*xy*	*I*_*y*_ *y*	*xy*
*f*_3_	*I*_*x*_ *y*	*I*_*y*_/*y*	*I*_*x*_ *x*	*xy*
*f*_4_	*I*_*y*_ *x*	*I*_*y*_/*y*	*I*_*x*_ *x*	*I*_*x*_ *y*

## Results and discussion

### Quantitative data and qualitative behavior

**Fig 3:**
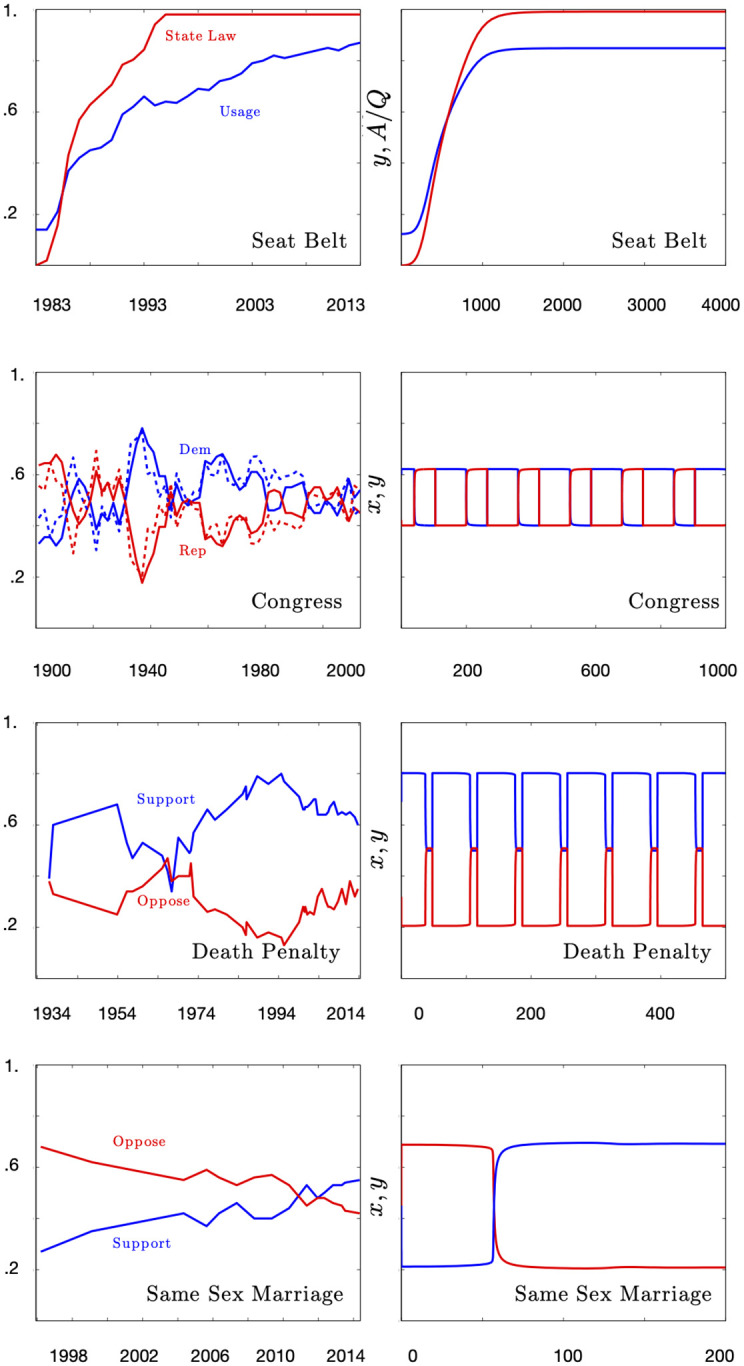
(Reading from left (empirical data) to right (simulated model) and top to bottom over the eight panels): Seat belt usage and number of states that have seat belt legislation paired with simulated results of model with fitted parameter values; Ratio of the US senate (solid line) and house (dotted line) seats for the Democrats and the Republicans and simulated model; Support and opposition of same-sex marriage and simulated model; Support and opposition of the death penalty and simulated model. Parameter values are provided in S2 File. In all of these cases the model is easily able to recover the qualitative features of the time series.

In order to provide intuition into a range of dynamics captured by the models for collectively encoded institutions through their public ledge values, consider the four following well known examples of institutional change (plotted in Fig 3):

1. Seat belt laws were first introduced in 1984 in NY state. Since then all state governments have introduced these laws and seat belt usage has significantly increased across the USA to a level in excess of 80%. [41]
2. A quintessential example of malleable institutions that wax and wane through time are political parties. Here we plot the ratio of US house and senate seats for the Democratic and Republican party. The patterns is one of ongoing, irregular, cycles. [42]
3. Official positions on same-sex marriage (SSM) have changed rapidly in the last few years with several institutions advocating on both sides of the issue. We observe in the data a rapid switch from opposition to support of SSM, largely as a result of successful advocacy campaigns and growing public awareness [19].
4. In the 1960’s there was a brief period when there were more opposing the death penalty (DP) than supporting it. The opinion never completely switched, and in the early 1990’s the support for DP reached mid the 80%. We find that the support/oppose ratio is slowly declining to around 60:40 [17].

Each of these time series can be qualitatively recovered through a suitable parametrization of the slow-manifold model and appropriate choice of learning rule (see S2 File). These are not technically ‘fits’ but qualitative regimes that reflect different choices of learning rule and parameter regime. The key learning insight is that periodic solutions are only compatible with rules that are not self-reinforcing.

Only the compensation rule and least effort rule can produce the switches and periodicity that we observe. The compensation rule describes an arms-race scenario where an agent invests in proportion to the success of its rivals. Thus when we consider attitudes to same sex marriage, or the death penalty, or seat belt use, by attending to the greater success of an idea that is opposed to one’s own, one’s idea grows in influence. Once this idea becomes dominant it is subject to complacency and liable to be replaced by the new minority view through the same negative frequency-dependent mechanism. The least-effort rule produces a similar dynamic but through a divestment of effort. Namely once dominant there appears little reason to continue learning and this allows the minority belief to learn its way into the majority. Political representation has just this character in which incumbent parties have no incentive to change and minimize their investment in supporting their constituency once they represent the majority. Variation across these plots is dominated by change in a single parameter, λ, which determines the half life of the agents. The shorter an agent lives the more likely the system is to generate periodic solutions.

**Fig 4:**
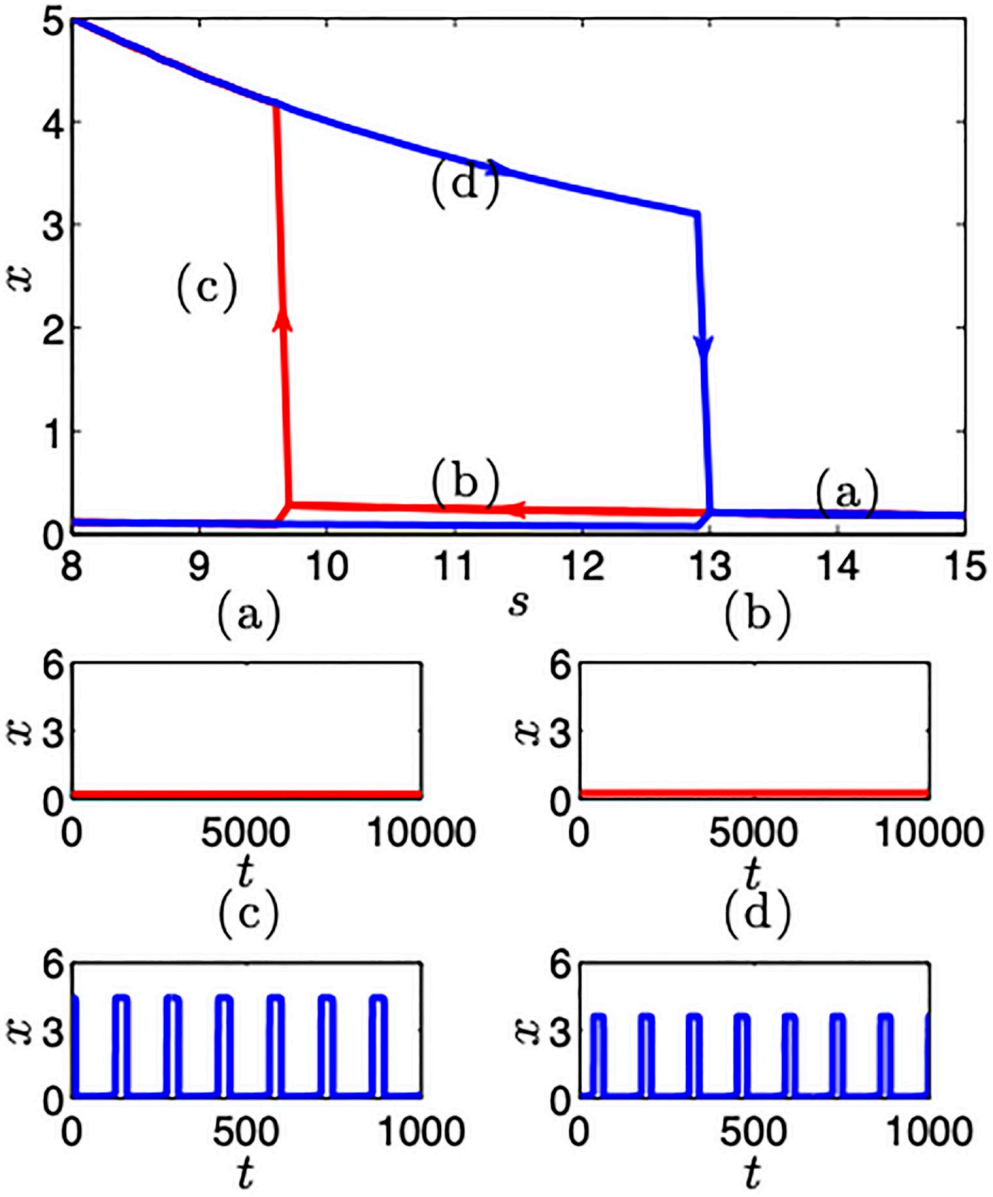
Learning rules generating institutional loss aversion–systemic endowment effect. Time series plot of *x* with *s* changing as *s* = 14(*a*)→11(*b*)→9(*c*)→11(*d*). In this case, once the model has reached a periodic configuration it remains so for higher values of *s* than when coming directly from a stable state. Institutions generate hysteresis producing an endowment-like effect whereby a state can be more readily acquired than relinquished. This maps onto the slow shifter class of collectively encoded institutional change.

Beyond these examples, we are able to enumerate the full range of model behaviors and analyze the stability of model solutions using linear stability analysis for the fixed points and Floquet theory for the periodic states (see S5 File). When we lower the value of the contagion/persuasion control parameter *s* the system undergoes a sub-critical Hopf bifurcation and settles into a periodic state. When *y* also employs a compensation rule, once in the periodic state, the system remains in this state for higher critical values of the control parameter. This phenomenon is known as *hysteresis* and arises from the different stability criteria for stable states and periodic states as shown in Fig 4. In this way feedback—effective downward causation [20] from institutions to agents—provides for a greater resistance to change—or loss aversion—between ‘effectively’ stable and periodic states. Agents can no longer change even when local mechanisms are strongly encouraging of change as a result of the feedback constraints of the institutions.

It can be useful to think about these regimes in qualitative terms. Apparent stasis with gradual nearly imperceptible surface change towards a new state as in seat belt adoption we call *Slow Shifter* institutions. *Tipping Point Like* institutions, by contrast, are marked by long stasis with apparent sudden change or reversals such as attitudes towards same sex marriage. Finally, the *Volatiles* are marked by persistent flipping or at least waxing and waning of support such as we see in the time series of political representation in congress and attitudes towards the death penalty. Each of these regimes corresponds to the adoption of a particular learning rule and parameter set. These classes of behavior are justified by the shape and curvature of a slow manifold.

### Slow manifold theory of institutions

How best to understand the relationship of microscopic agents to collectively encoded macroscopic institutions? And how do the time scales of agent-agent-interaction, agent-institution construction, and agent learning be understood within a single analytical framework of institutional change? We need to account for the possibility that periods of stasis are punctuated by periods of rapid change (Tipping Point Like institutions) without equilibrium being attained and yet also allow for an underlying dynamic that leads to institutions that change rapidly (Volatile institutions) or change gradually with no reversals (Slow Shifter institutions).

We make progress with the analysis of these systems by recognizing that they fall into the category of ‘fast-slow dynamics’ with respect to the microscopic-macroscopic relationship [43]—systems of the form:

$$\begin{array}{c} \frac{dz}{dt}\;=f(z,w,\varepsilon ), \end{array}$$ (6)

$$\begin{array}{c} \frac{dw}{dt}\;=\varepsilon g(z,w,\varepsilon ), \end{array}$$ (7)

where $z\in R^{2}$ vector of fast individual variables (*x*, *y*), $w\in R^{n}$ vector of slow institutional variables, where *ε* is a parameter representing the separation between the time scales of agent behavior and agent learning.

### Classification of behaviors under different learning rules

There are five models that fit into a category in which we track one fast (*y*) and two slow (*w*_1_ and *w*_3_ or *w*_4_) variables by recognizing that $x+y=\hat{r}$ (S1 File). A prototypical system of this sort in two dimension is the van der Pol oscillator. In our case, these models become:

$$\begin{array}{c} \frac{dw_{1}}{dt}\;=\varepsilon g_{1}\left(w_{1},w_{3},y\right)\;, \end{array}$$ (8)

$$\begin{array}{c} \frac{dw_{3}}{dt}\;=\varepsilon g_{3}\left(w_{1},w_{3},y\right)\;, \end{array}$$ (9)

$$\begin{array}{c} \frac{dy}{dt}\;=f(w_{1},w_{3},y)\;, \end{array}$$ (10)

where $f\left(w_{1},w_{3},y\right)=\left[s\left(I_{y}+q\right)\left(\hat{r}-y\right)y-\lambda_{y}-cI_{x}\right]y$. The two-dimensional surface—the slow manifold—is given by

$$\begin{array}{c} S=\{\left(w_{1},w_{3},y\right)\in R_{+}^{3}:f\left(w_{1},w_{3},y\right)=0\} \end{array}$$ (11)

This slow manifold is separated by fold curves into three different regions. The upper and lower regions are attracting planes ($\frac{\partial f}{\partial y}<0$) while the middle regions is repelling ($\frac{\partial f}{\partial y}>0$). The fold curves is given by

$$\begin{array}{c} L^{\pm }=\left\{\left(w_{1},w_{3},y\right)\in S:\frac{\partial f}{\partial y}\;=0\right\} \end{array}$$ (12)

**Fig 5:**
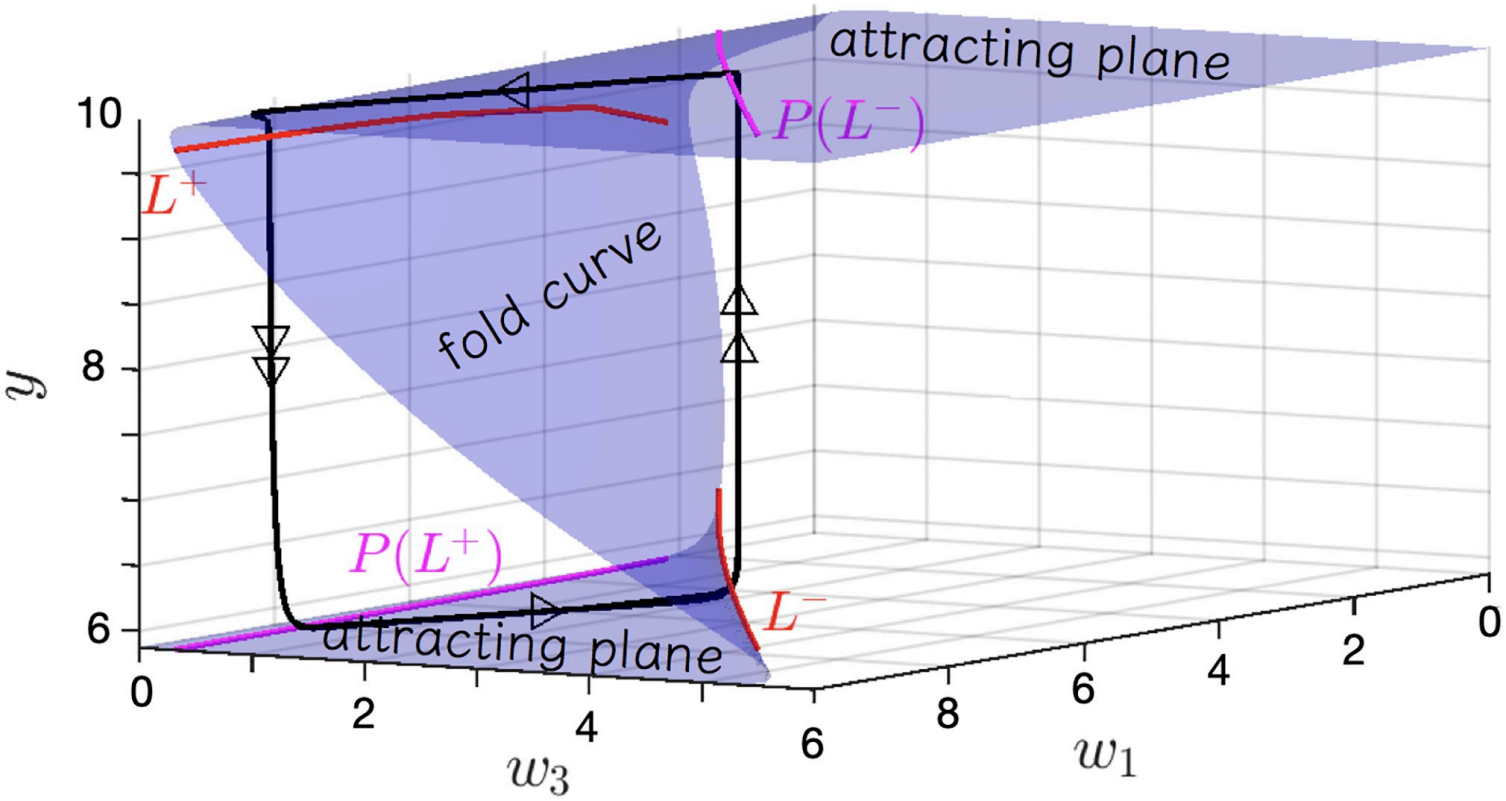
The slow manifold of institutional change. The vertical axis *y* is the abundance of agent *y* (we could as easily have shown *I*_*y*_ which are simple mappings from *y* and *x*). The learning axes, *w*_1_ and *w*_3_ capture the learned effort invested by populations of agents *x* in supporting their own institution through their public ledge values *I*_*x*_ while opposing rival institutions and their position values *I*_*y*_. The dynamical system remains tethered to this 2-dimensional plane such that when the learning parameters cross certain threshold values a sudden change in the abundance of *y* is observed. High values of *w*_1_ and low values of *w*_3_ are associated with low values of *y* and high values of *x*. The explanation for the shape of this manifold derives from the relative rates of change of agent abundance versus learning and hence the institutional time scales. The flat portion of the manifold reflects the quasi-stationary solutions of the fast dynamics for a given range of values of *w*_*i*_. The folds of the manifold are traversed when these slow learning parameters reach a critical ‘transmission’ threshold with respect to the faster agent scale dynamics in *x* and *y*. In this way the folds of the manifold appear to be equilibrium solutions to a low dimensional system whereas they are in fact flat portions of trajectories in a high dimensional space.

The slow manifold is shown in Fig 5. All possible combinations of learning rules and their detailed stability results are provided in S6 File. The slow shifter corresponds to a graduated manifold in which motion along the *w*_*i*_ axes is accompanied by proportional motion along the *y* axis; tipping points are produced by flat manifolds; and volatiles by small attracting planes in which short distances on the plane lead to rapid movement off the fold curve.

## Conclusions

Institutions are regulatory mechanisms acting on collective dynamics and as such enable both large-scale control and memory [44]. Institutional dynamics, by virtue of having multiple timescales [20], can temporarily stabilize individual preferences and behaviors and, over longer periods of time, facilitate social change [45, 46]. In the case of institutions, the timescales include (1) a fast timescale of direct competition among individuals seeking to record preferences on institution formation, (2) a slow time scale involving learning wherein individuals adapt to (3) the slowest time scale of institutions. These multiple timescales support feedback from the average or ‘macroscopic’ outputs of collective behavior to the individuals constituting ‘microscopic’ inputs to society [20]. This third timescale, corresponding to the rate of surface change, is an emergent feature of dynamics rather than an explicit “hand-coded” property of a model. An important goal of this paper has been to identify the underlying dynamics responsible for different types of collectively encoded institutions–volatiles, slow shape shifters and tipping point like institutions. In this respect this paper is largely an effort toward synthesis bringing insights from non-linear dynamics to bear on questions of social and cultural change.

To allow agent-institution interaction, we’ve operationalized collectively encoded institutions as private ledgers with public positions connected within a network of learning agents. Agents cast positive and negative votes. Overtime these votes are summed to record the cumulative collective preference of populations of competing agents. These positions can be thought of as recording the weight of *opinion* favoring a given doctrine, idea, or social norm. It would be interesting to represent the ledger more fully as a vector encoding the entire history of exchange. Through such an extended model we could establish some correspondence with explicit institutional ledgers such as the blockchain which not only allows for transaction but for verification. Recently the use of news aggregation sites on the web has instantiated literal digital containers of public opinion supporting both consensus formation scores and a tracking of histories contributing to new forms of collective herding dynamics [47].

Given learning is ongoing the votes in the position never reach an equilibrium value and so change at the agent-level becomes inevitable. By introducing multiple timescales we observe a ‘slow manifold’ which can produce short-term-stasis, and, in the longer-term, yield rapid change that epitomizes the tipping point like surface change that seems to be typical of institutions like scientific ideas.

The key insight of the slow manifold is that it shows how in high dimensional dynamical systems, many fast processes can remain hidden, and give the appearance of a low dimensional system dominated by slow processes. Moreover, what look like fixed points are only quasi-stationary states and given enough time, invisible fast dynamics can cause a system to undergo very rapid transitions. It is these properties of the slow manifold that makes institutional dynamics special and somewhat different from typical population dynamics. These dynamics could be expanded by examining the far larger set of transmission and contagion dynamics which contain SIR-type models as a special case. The current framework does not capture instances where interactions between agents are symmetric—such as when agents share information equally- or cases where effective transmission depends on higher order network interactions.

We find the choice of local-learning rules by agents strongly determines global dynamics and can shift the system away from tipping point surface change. Reinforcement dynamics that amplify absolute success freeze-in dominant agents and their institutional preferences (permanent stasis). These dynamics might underlie the slow shifter institutions like political doctrines as discussed in the Introduction with respect to core properties of liberalism and the death penalty. A novel prediction of these models is that resource-minimizing rules which expend effort in response to relative success (compensation), promote cycles of stasis and rapid change. These dynamics perhaps underlie volatile institutions like united states political party dominance and possibly support for the filibuster. A second prediction of these models is that learning networks using resource minimizing rules experience hysteresis- in which there is a strong directional preference for either increases or decreases in position values. This leads to a new explanation for endowment effects in terms of the collective dynamics of learning networks in distinction to the more typical individual aversion to change. The key idea is that learning rules act in an analogous way to control parameters and thereby provide a means of modulating global institutional dynamics using only local reward signals.

Several hypotheses have been proposed in the literature to explain economic, political, scientific, and institutional change. Of significant historical importance are the ideas of Compte, Durkheim and Marx, seeking to explain progressive and directed biases [48], rationality, role differentiation, connectivity, equality, and solidarity [49]. All of these rely implicitly or explicitly on individual adaptation and learning, and all allow for the appearance of multiple equilibria at the societal scale. The common denominator for most of these theories is the neo-darwinian theory of evolution through mutation (noise), drift (sampling) and selection (reinforcement), to include extensions for hierarchical selection [50] and niche construction [29].

Within the evolutionary community itself, growing importance has been placed on taking into account feedback through effective downward causation from consolidating levels of organization [20, 51], from more inclusive levels of selection (hierarchical selection theory: [50]), and long-term contributions of organismic behavior to environmental features through niche construction [29] and ecosystem engineering [52]. The latter two frameworks–niche construction and levels of selection theory–have tended to rely on equilibrium solutions (infinite-time approximations) and are less focused on change than they are on novel or unexpected distributions of strategies (e.g. cooperation and related phenomena such as reproductive altruism, eusociality, and Quorum sensing [53]). In this study the equilibrium concept is abandoned in favor of the theory of the slow manifold traversed via learning with an emphasis on connecting long-term cycles of change with work on collective computation of slow variables and downward causation [20, 51, 54].

Whereas neoclassical economics historically tended to downplay multiple equilibria, there have been long-standing calls for a framework that might allow for ‘moving equilibria’ [55], which has come to be supported by modern game theory [56] through which a number of issues relating to changing social norms have been investigated [57]. The more recent fields of institutional economics and comparative economics have explicitly built on ideas related to Darwinian frameworks, and allow for a more comprehensive approach to change. Notable among these approaches to institutions is the work of Nelson and Winter ([58]), Fogel and North ([2]), and Acemoglu and Robinson ([59]). Like the modeling framework introduced in this paper, these projects emphasize local rules and learning coupled to emergent institutional constraints rather than global maximization and stable fixed-points.

In the historical setting of scientific change, the space of ideas is bounded by Popperian anomalies, Lakatosian bookkeeping, Kuhnian paradigm changes, and Feyerabendian anarchy [60]. All agree that scientific change is in the long-term promoted through irreconcilable difference between observation and theoretical expectation: operational and instrumental ideas carry the day in the ‘longue duree’. All differ on the rapidity of adoption of key refuting evidence and the relative role of social factors over hypothetico-deductive empiricism. From the perspective of this study these differences of opinions can be treated as simple differences in the relative importance of agent-agent interactions and feedback from institutional position values. Naive Popperian refutation requires only a single persuasive agent-agent interaction. Lakatosian change is realized almost exclusively through lists of ledger values whose sum is their position that feeds-back to agents. The Kuhnian paradigm–as well as the framework introduced by Acemoglu and Robinson–comes closest to the models presented here: fast agent interactions (transmission) and slow positional updates through suitable learning rules promoting cycles of normal and revolutionary activity. However, as we have noted, the pattern of change at the institutional level can be shifted from tipping point like institutional change to slow shifter change or volatile change by changing learning rules without requiring exogenous perturbation or fixed points, thereby allowing us to explore a wide range of institutional change within a single modeling framework. In this respect, our framework deviates from Kuhn.

Future work should consider the relative role of slow shifter institutions generated by hysteresis versus the resistance to change at the individual level in contributing to large-scale endowment effects [61]. There is also the open question of the effect of technology on human learning networks. Technology increases rates of transmission. Fast transmission with slow learning tends to amplify the separation of time scales and militate against institutional change. An open set of questions therefore relates to how technology interfaces with institutional dynamics to promote stasis or change. Finally we would like to acquire further evidence from systems that appear to have reached a steady state but are in fact near a critical learning threshold. Such as those present in positive popular opinion towards gay marriage and are very likely to have been a latent characteristic of the historic Black Lives Matter protests of 2020.

## Supporting information

S1 File Institution independent dynamics. (PDF)

S2 File Parameters for empirical case studies. (PDF)

S3 File Exploring the implications of static versus dynamical institutions. (PDF)

S4 File Properties of multiple time scales. (PDF)

S5 File Enumeration of full rule space and stability properties. (PDF)

S6 File Properties of the slow manifold. (PDF)

S7 File Monopolar institutions. (PDF)
