# Supplementary material for: Institutional dynamics and learning networks
Source: PLoS One. 2022 May 16;17(5):e0267688. doi: 10.1371/journal.pone.0267688 (PMC9109929; doi:10.1371/journal.pone.0267688)
Supplement: S1 File — (PDF) [file pone.0267688.s001.pdf]

S1 File. The Institution independent dynamics

The basic infection dynamics between two different populations of agents x and y without institutional input is described through the following system of differential equations:

$$\frac{dx}{dt} = r - sxy - \lambda_x x \; ; \tag{1}$$

$$\frac{dy}{dt} = sxy - \lambda_y y \; ; \tag{2}$$

where r, s, λ_x and λ_y are all positive parameters. This system has a stable equilibrium solution and it is

$$\left(\text{For } \frac{rs}{\lambda_x \lambda_y} > 1\right): \qquad \hat{x} = \frac{\lambda_y}{s}, \quad \hat{y} = \frac{r}{\lambda_y} - \frac{\lambda_x}{s} \; . \tag{3}$$

or

$$\left(\text{For } \frac{rs}{\lambda_x \lambda_y} \leq 1\right): \qquad \hat{x} = \frac{r}{\lambda_x}, \quad \hat{y} = 0 \; . \tag{4}$$

This equation is often used to capture average properties of transmission processes. The value of $R_0 = \dfrac{rs}{\lambda_x \lambda_y}$ (the basic reproductive ratio) dictates whether sustained tranmission occurs.

The steady state solution (3) can be modified by adjusting the value of key parameters, and by doing so, decrease the abundance of one strategy and increase the value of the opposing strategy. For example, a larger value of s will lead to a smaller $\hat{x}$ and larger $\hat{y}$.

Assume that $\lambda = \lambda_x = \lambda_y$, we can non-dimensionalize the system (1)–(2) with

$$x = \frac{\lambda}{s} u; \quad y = \frac{\lambda}{s} v; \quad t = \frac{T}{\lambda}; \quad \hat{N} = \frac{rs}{\lambda^2}. \tag{5}$$

and (1)–(2) becomes

$$\frac{du}{dT} = \hat{N} - uv - u \; ; \tag{6}$$

$$\frac{dv}{dT} = uv - v \; . \tag{7}$$

Note that if $v(0) > 0$ the system will reach the steady state solution $\hat{u} = 1, \hat{v} = \hat{N} - 1$. In fact, the solution for $N(T) = u + v$ is

$$N(t) = \hat{N} - (\hat{N} - N(0)) \exp(-T) \; , \tag{8}$$

which approaches $\hat{N}$ for large T. Using the same choice of variables as (5) we can rewrite (**??**)–(**??**) as

$$\frac{du}{dT} = \hat{N} - (I_y + q)uv - u + rI_x v \; ; \tag{9}$$

$$\frac{dv}{dT} = (I_y + q)uv - v - rI_x v \; , \tag{10}$$

where $r = \frac{c}{\lambda}$. If I_y and I_x are constant, then the stationary solutions u and v are $\hat{u} = \dfrac{1 + rI_x}{I_y + q}, \hat{v} = \hat{N} - \hat{u}$. Same result holds for a single institution model, where agents alternately construct and deconstruct, by letting $I_x = 0$. Note that letting $\lambda_x \neq \lambda_y$, then the stationary solutions of (1)–(2) becomes $\hat{u} = \alpha, \hat{v} = \dfrac{\hat{N}}{\alpha} - 1$, where $\alpha = \dfrac{\lambda_y}{\lambda_x}$. This implies that $x + y$ is not a conserved quantity.
