# Supplementary material for: Institutional dynamics and learning networks
Source: PLoS One. 2022 May 16;17(5):e0267688. doi: 10.1371/journal.pone.0267688 (PMC9109929; doi:10.1371/journal.pone.0267688)
Supplement: S2 File — (PDF) [file pone.0267688.s002.pdf]

S2 File. Parameters for empirical case studies

The following table describes the parameter values chosen to mimic the qualitative behavior of the four empirical data sets. We are not interpreting these values as particularly meaningful other than as a means of illustrating the range of dynamical behaviors produced by the single model. The key variable controlling the different qualitative regimes is the half life of each agent governed by the λ values. High rates of agent turnover (high λ) promote periodic solutions.

Example	Model	Learning Rule(s)	s	$\lambda_{x,y}$	r	q	Q	ε	c
Seat Belt	One-Institution	Compensation	1	1	1	1	0.005	5	/
Death Penalty	Two-Institution	Compensation $\times$ 2	4	0.45	10	0.05	0.02	1	4
Same-sex Marriage	Two-Institution	Compensation $\times$ 2	4	0.625	10	0.05	0.02	1	1
Congress	Two-Institution	Least effort $\times$ 2	4	1	10	0.05	0.02	1	1

Table 1. Qualitative dynamics under different learning rules.
