# Supplementary material for: Institutional dynamics and learning networks
Source: PLoS One. 2022 May 16;17(5):e0267688. doi: 10.1371/journal.pone.0267688 (PMC9109929; doi:10.1371/journal.pone.0267688)
Supplement: S3 File — (PDF) [file pone.0267688.s003.pdf]

S3 File. Exploring the implications of static versus dynamical institution

In this section we consider a candidate dynamical approach (differential equations) in contrast to separation of timescales considered in the paper (functional equations) for modeling institutional change. This is equivalent to allowing the construction of institutions to approximate the time scales of learning. This might correspond to cases where there is an additional time scale delaying the change of an institution above and beyond the time scale of individual learning that drives the change. For example a latent regulatory delay on the adoption of a new policy, or a delay in the publication of a widely held idea. In the manuscript our assumption is that these time scales will typically be much shorter than the time required for individual adoption and diffusion of ideas and policies.

Note that we are modeling the *dynamics* of the institutions via the adaptive weights $\vec{w}$, rather than treating I_y and I_x as additional variables in the dynamical system. One of several ways in which we might accomplish this through,

$$\frac{dI_x}{dt} = \phi(w_1 x - w_3 y) - mI_x \; ; \tag{1}$$

$$\frac{dI_y}{dt} = \phi(w_4 y - w_2 x) - mI_y \; , \tag{2}$$

where m establishes the time scale at which I_x and I_y relax to ϕ. Figure 1a and 1b shows the dynamics of the variables for $m = p$ and $m = 10p$. In the first case, the dynamics of I_x and I_y are on the same scale as $\vec{w}$, producing a resonance effect on x and y. In the second case, the dynamics of I_x and I_y are faster than $\vec{w}$ and slower than x and y, and there is little change to the overall behavior of the system. Note that in the manuscript we are assuming that $\frac{dI_x}{dt} = \frac{dI_y}{dt} = 0$, that is the institutions reach equilibrium quickly. With this fast-slow dynamics there is the added a prefactor $1/m$ that rescales the value of the public position. A full exploration of a dynamical approach could involve a large variety of alternative models. This could even be extended to include high level learning-rules that modify institutions based on the perception of rival institutions – that is through direct institutional coupling. This constitutes further work beyond the assumptions of this contribution.

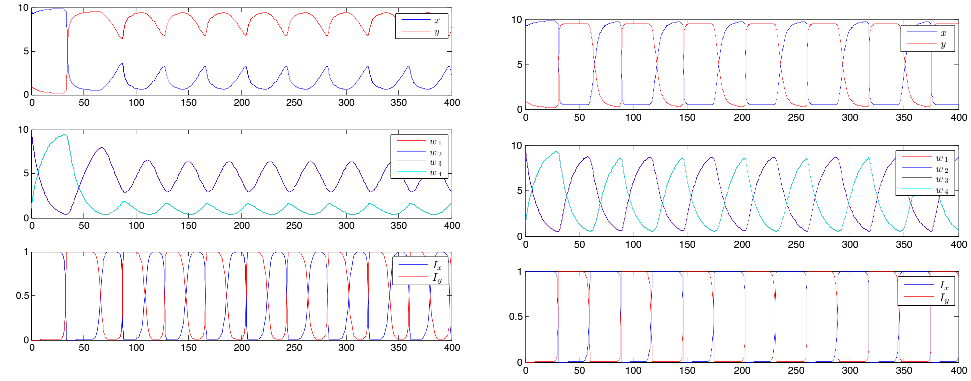

(a) **(b)**

Fig 1. Figures for SI 3: Solutions of x, y, I_x, I_y and $\vec{w}$ under the compensation rule with the dynamics of I_x and I_y represented by (1) and (2). In (a): $m = p = k = 0.01$ and in (b), $m = 10p = 10k = 0.1$.

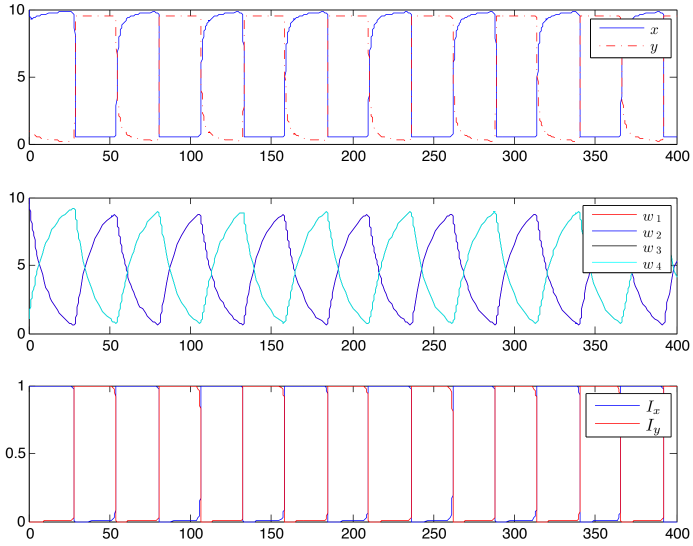

Fig 2. Figures for SI 4: Solutions of x, y, I_x, I_y and $\vec{w}$ under the compensation rules with the two institution model. Here, $p = k = 0.01$.
