# Supplementary material for: Institutional dynamics and learning networks
Source: PLoS One. 2022 May 16;17(5):e0267688. doi: 10.1371/journal.pone.0267688 (PMC9109929; doi:10.1371/journal.pone.0267688)
Supplement: S4 File — (PDF) [file pone.0267688.s004.pdf]

S4 File. Properties of multiple time scales

From supplemental information S3 we observe the time series of x, y, I_x, I_y and $\vec{w}$ in the dual-institution model under the Competition rule. The behavior of the variables can be summarized as follows: The dynamics of x and y are characterized by relatively slowly changing periods of length $O(\varepsilon^{-1})$, during which they remain either close to an equilibrium state (probable state) of the microscopic model or maximally distant (improbable state). Between these states, there are rapidly changing periods of length $O(1)$ during which x and y switch from one state to another. These long quasi-stationary periods alternate with short periods that coincide with dynamics of $\vec{w}$ and the fast switches in the institutional abundances I_x, I_y. By adjusting the ratio p to k, the slow time-scale parameters in the equations for $\vec{w}$, allow us to tune the ratio of the duration of stasis between states. This connects to the idea of tuning a system to a critical point where change is likely versus a state which is relatively stable and thereby robust to changes in parameter values. This provides a way of thinking about the historicity of institutions and provides a clue as to the most effective points of interventions into the dynamical system. We note that the symmetry of the learning rules lead to the synchronization of the weight variables $\vec{w}$, since when $f_i = f_j$ then $\dfrac{d(w_i - w_j)}{dt} = -p(w_i - w_j)$, which implies that $w_i - w_j = O(e^{-pt})$. In this example, $f_1 = f_2$ and $f_3 = f_4$.
