# Supplementary material for: Institutional dynamics and learning networks
Source: PLoS One. 2022 May 16;17(5):e0267688. doi: 10.1371/journal.pone.0267688 (PMC9109929; doi:10.1371/journal.pone.0267688)
Supplement: S5 File — (PDF) [file pone.0267688.s005.pdf]

S5 File. Enumeration of full rule space and stability properties

This section enumerates all possible solution concepts and stability results for each combination of paired learning rule. The signs of the partial derivatives required for stability are indicated with the symbols, $+,-$. Fast and slow refer to the relative magnitudes of the relevant rate constants in the dynamical system. For example in the 'one fast two slow' condition we have one fast agent variable y and two learning rate variables w_1, w_2. And the description in terms of periodic orbits or fixed points the results of linear stability analysis interrogating the signs of the dominant eigenvalues of the stability matrix.

One fast two slow model (red and Magenta)

1. Compensation–Compensation Periodic Orbit with hysteresis.
2. Compensation–Least Effort Period Orbit, Canards and mixed-mode oscillation.
3. Least Effort–Compensation One fixed point is a saddle (+,-,-), one fixed point is stable (-,-,-).
4. Least Effort–Least Effort No strict equilibrium.
5. Least Effort–Competition One fixed point is a saddle (+,-,-), one fixed point is stable (-,-,-).
6. Hebbian–Compensation Both fixed point are saddles–(+,-,-) and (+,+,-).

One fast three slow model (Green)

1. Compensation–Competition Periodic Orbit, Hysteresis with relaxation oscillation at a second region.
2. Compensation–Hebbian Periodic Orbit, Canard, Possible Homoclinic Orbit.
3. Least Effort–Hebbian One fixed point is a saddle (+,-,-,-) two fixed points are stable (-,-,-,-).

One fast three slow model (Blue)

1. Hebbian–Least Effort One fixed point is stable (-,-,-,-).
2. Competition–Least Effort No strict equilibrium.
3. Competition–Compensation One fixed point is stable (-,-,-,-).

One fast three slow models

1. Competition–Competition One fixed point is stable (-,-,-,-).
2. Competition–Hebbian Two fixed points are stable (-,-,-,-).
3. Hebbian–Hebbian One fixed point is stable (-,-,-,-).

Summary

Rule by x / Rule by y	Least Effort	Compensation	Competition	Hebbian
Least Effort	PO	PO	PO	SE
Compensation	SE, HO	PO	SE	EO
Competition	SE, HO	PO	SE	HO
Hebbian	SE, HO	PO	SE	SE

Table 1. Table of various type of behavior using different sets of learning rules (SE = Stable Equilibria, PO = Periodic Orbit, HO = Homoclinic Orbit, EO = Heteroclinic Orbit
