# Supplementary material for: Institutional dynamics and learning networks
Source: PLoS One. 2022 May 16;17(5):e0267688. doi: 10.1371/journal.pone.0267688 (PMC9109929; doi:10.1371/journal.pone.0267688)
Supplement: S6 File — (PDF) [file pone.0267688.s006.pdf]

S6 File. Properties of the slow manifold

Rule by y \ Rule by x	Least Effort	Compensation	Competition	Hebbian
Lease Effort	$w_1 = w_2, w_3 = w_4$ (3)	$w_1 = w_2, w_3 = w_4$ (3)	$w_3 = w_4$ (4)	$w_3 = w_4$ (4)
Compensation	$w_1 = w_2, w_3 = w_4$ (3)	$w_1 = w_2, w_3 = w_4$ (3)	$w_3 = w_4$ (4)	$w_1 = w_3 = w_4$ (3)
Competition	$w_1 = w_2 = w_3$ (3)	$w_1 = w_2$ (4)	$w_2 = w_3$ (4)	None (5)
Hebbian	$w_1 = w_2$ (4)	$w_1 = w_2$ (4)	$w_1 = w_4$ (4)	$w_2 = w_4$ (4)

Table 1. Symmetry and number of dynamical variables (in bracket) in the two-institution model with distinct sets of learning rules adopted by x and y. Different colors indicates models in the same category. We concentrate in the paper on the one fast and two slow model illustrated by the red and magenta entries.

When y employs a different learning rule from x there is no hysteresis in the bifurcation. To understand this formally, one need consider that at the fold curve the critical manifold loses its hyperbolicity (real part of the eigenvalue becomes zero), causing the geometric singular perturbation theory to break down. However, one can rewrite the dynamics by re-scaling and obtain a new equation of motion for the de-singularized system:

$$\frac{dy}{d\tau} = g_{xx}\frac{\partial f}{\partial w_{xx}} + g_{yx}\frac{\partial f}{\partial w_{yx}} \ ,$$
$$\frac{dw_{xx}}{d\tau} = g_{xx}\frac{\partial f}{\partial y} \ .$$

where $\tau = -(\frac{\partial f}{\partial y})^{-1}$, w_{yx} satisfies $f(w_{xx}, w_{yx}, y) = 0$. This is derived by differentiating $f(w_{xx}, w_{yx}, y) = 0$ with respect to t. The fold node (red dot in 1) is the fixed point of this system: a saddle note with one positive and one negative eigenvalue.

Fig. 1 illustrates the two different behaviors. There is a small-scale mixed mode oscillation (inset) with periodicity sitting between the fast time scale t and the slow time scale εt. This is followed by a canard in which y and w_{xx} are temporarily sitting on the unstable manifold before they are kicked back onto the critical manifold.

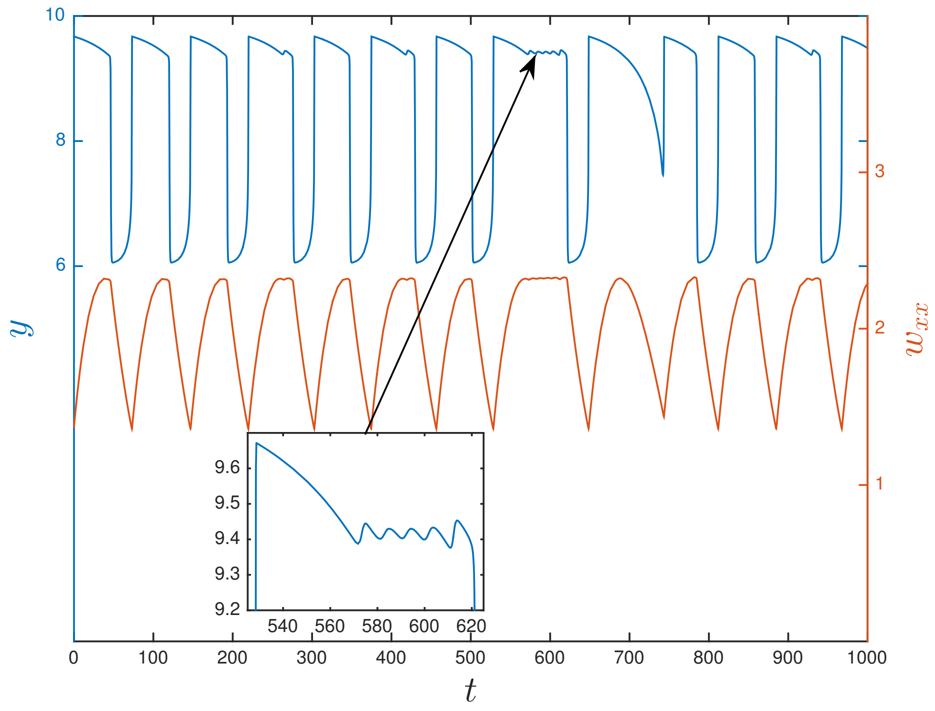

Fig 1. For SI6: Canards or very fast transitions sometimes called canard explosions taking place within an exponentially small range of a control parameter.
