# Supplementary material for: Institutional dynamics and learning networks
Source: PLoS One. 2022 May 16;17(5):e0267688. doi: 10.1371/journal.pone.0267688 (PMC9109929; doi:10.1371/journal.pone.0267688)
Supplement: S7 File — (PDF) [file pone.0267688.s007.pdf]

S7 File. Monopolar Institutions

One can conceive of a simplified model in which there is only *one* institution.

$$\frac{dx}{dt} = r - s(I_y(x,y,\gamma_1,\gamma_2)+q)xy - \lambda_x x \; ; \qquad (1)$$

$$\frac{dy}{dt} = s(I_y(x,y,\gamma_1,\gamma_2)+q)xy - \lambda_y y \; ; \qquad (2)$$

$$(3)$$

where

$$I_y(x,y,\gamma_1,\gamma_2) = \phi(\gamma_2 y - \gamma_1 x) \; ; \qquad (4)$$

$$\phi(z) = \frac{Q \exp z}{\exp z + Q} > 0 \, . \qquad (5)$$

The effective transmissibility in (1)–(2) is still $s(I_y+q)$–we have essentially dissect the impact of y on x into parts that is either independent of I_y ($sqxy$ term represents the baseline interaction if there is no institution I_y) and scaled with I_y (sI_yxy). When $I_y > 1(<1)$, the density of y (x) increases. In turn, I_y is built on y (as y becomes large, $I_y \to Q$) and is suppressed by x (as x becomes large, $I_y \to 0$). The weight variables $\vec{\gamma}$ that determine the size of contribution of x and y to I_y is modeled by the following equation

$$\frac{d\gamma_i}{dt} = kg_i(I_y,x,y) - p\gamma_i, \qquad (6)$$

where $k \approx p \sim \varepsilon \ll 1$. In other words, the weight variables evolve in the *slow* time scale $T = \varepsilon t$. The different functions $g_i(\cdot)$ provide positive feedbacks to γ_i according to the state of the system.
